# Plant arginyltransferases (ATEs)

**DOI:** 10.1590/1678-4685-GMB-2016-0084

**Published:** 2017-02-13

**Authors:** Tatiana Domitrovic, Anna K. Fausto, Tatiane da F. Silva, Elisson Romanel, Maite F. S. Vaslin

**Affiliations:** 1 Laboratório de Virologia Molecular Vegetal, Departamento de Virologia IMPPG, Universidade Federal do Rio de Janeiro, Rio de Janeiro, RJ, Brazil; 2 Departamento de Biotecnologia, Escola de Engenharia de Lorena, Universidade de São Paulo, Lorena, SP, Brazil

**Keywords:** ATE, N-end rule, plant, argyniltransferase, protein degradation

## Abstract

Regulation of protein stability and/or degradation of misfolded and damaged proteins are essential cellular processes. A part of this regulation is mediated by the so-called N-end rule proteolytic pathway, which, in concert with the ubiquitin proteasome system (UPS), drives protein degradation depending on the N-terminal amino acid sequence. One important enzyme involved in this process is arginyl-t-RNA transferase, known as ATE. This enzyme acts post-translationally by introducing an arginine residue at the N-terminus of specific protein targets to signal degradation via the UPS. However, the function of ATEs has only recently begun to be revealed. Nonetheless, the few studies to date investigating ATE activity in plants points to the great importance of the ATE/N-end rule pathway in regulating plant signaling. Plant development, seed germination, leaf morphology and responses to gas signaling in plants are among the processes affected by the ATE/N-end rule pathway. In this review, we present some of the known biological functions of plant ATE proteins, highlighting the need for more in-depth studies on this intriguing pathway.

## Introduction

Protein post-translational modifications are responsible for biological regulation of many important physiological processes and are the focus of intense research. However, the cellular functions associated with certain protein modifications described many years ago are only recently being revealed. This is the case for the incorporation of Arg residues into proteins by a mechanism that is dependent on tRNA but independent of conventional translation. Discovered in 1963 by Kaji *et al*. (1963a,b), the modification known as arginylation occurs in some bacteria and all eukaryotic organisms and is mediated by the enzyme arginyltransferase (ATE). *ATE* genes are found in organisms ranging from yeasts to humans. The enzyme is encoded by a single gene in lower eukaryotes and by multiple isoforms in higher eukaryotes (Kwon *et al*., 1999; Hu *et al*., 2005; Rai and Kashina, 2005). In this short review we summarize some of the knowledge about plant ATEs, highlighting the biological process in which they modulate protein stability. Although Manahan and App (1973) first identified a plant arginyltransferase, approximately 30 years elapsed before further details about the cellular processes regulated by this modification emerged.

Knockouts of yeast and *Caenorhabditis elegans ATE1* genes give rise to viable organisms with no obvious phenotypes, whereas *ATE1* deletion in *Drosophila melanogaster* and *Mus musculus* results in embryonic lethality (Kwon *et al*., 2002; Crowe and Candido, 2004; Mutsuddi *et al*., 2004). Lee *et al*. (2012) showed that *ATE1*-knockout mouse embryos present cardiovascular and angiogenesis defects. These observations suggest that arginylation has acquired new functions and more specialized and essential roles in higher eukaryotes.

In plants, however, ATE is not required for viability (Yoshida *et al*., 2002; Graciet *et al*., 2009; Holman *et al*., 2009). Regardless, as presented in the following sections, *Arabidopsis* ATE isoform mutants have revealed important phenotypes associated with seed germination and plant development, suggesting that this post-translational modification is involved in diverse regulatory functions in eukaryotes.

## Chemistry of arginylation and the N-end rule pathway of protein degradation

Arginyltransferases from eukaryotes and the related family of L/F transferases from bacteria are the only known class of enzymes that can transfer amino acid residues from aminoacyl-tRNA to proteins in a way that is independent of ribosomal protein synthesis (Kaji *et al*., 1963a,b, 1968; Bulinski, 2009). Studies with partially purified ATE preparations, and more recently with recombinant mouse ATE isoforms, have revealed that ATE does not require any factors other than the target protein and charged tRNA to catalyze arginine transfer (Wang *et al*., 2011). Proteins with N-terminally exposed Glu or Asp residues are typical ATE targets. The reaction is thought to result in a peptide bond between the alpha amino group of the N-terminal residue of a protein and the carboxy group of the added Arg that becomes the first residue (Kaji, 1968). However, recent studies have demonstrated that arginylation is not restricted to the N-terminus of proteins but can also occur at internal Asp and Glu side chains, adding a new layer of complexity to the reaction (Wang *et al*., 2011, 2014; Wong *et al*., 2007). It was also demonstrated that ATE catalyzes self-arginylation and that different mouse ATE isoforms exhibit different kinetic properties and substrate specificity, which suggests that the differential expression of ATE variants is important for target selectivity (Wang *et al*., 2011).

The functional implications of arginylation accompanied the observation that proteins with an N-terminally exposed Arg or arginylated proteins are unstable in the cytosol of cells from different organisms (Bachmair *et al*., 1986; Gonda *et al*., 1989). Furthermore, it was shown that cellular degradation of proteins with an acidic N-terminus is dependent on arginylation and on the ubiquitin proteasome system (UPS) (Elias and Ciechanover, 1990). Therefore, arginylation is now considered an important aspect of the so-called N-end rule pathway of protein degradation.

The N-end rule pathway relates to a series of orderly reactions that control protein degradation depending on the nature of the N-terminal residue (Bachmair *et al*., 1986). The presence of the classic destabilizing amino acids Arg, His and Lys at the N-terminus of a protein is a degradation signal, or degron, that is recognized by a specific type of E3 ubiquitin ligases, the N- recognins, called proteolysis protein or PRT in plants (Potuschak *et al*., 1998; Stary *et al*., 2003) and ubiquitin system recognition component or UBR in animals (Sriram *et al*., 2011). In association with the destabilizing residues, the target protein must have an optimally positioned downstream Lys residue as a site for polyubiquitination and an appropriate secondary and/or tertiary structure (Tasaki *et al*., 2009, Varshavsky, 2011; Kim *et al*., 2013). E3 ubiquitin ligases direct degron-containing proteins toward degradation via the 26S proteasome (Tasaki *et al*., 2012).

The basic amino acid residues Arg, His, and Lys as well as hydrophobic Phe, Trp, Tyr, Leu, and Ile residues are called primary destabilizing amino acids because their presence at the N-terminus of a protein is a determinant for proteolysis. Asp, Glu or oxidized Cys are ATE substrates, and the protein may become a substrate for E3 ligases following arginylation. Therefore, these amino acids are known as secondary destabilizing residues. Gln, Asn and Cys are considered tertiary destabilizing amino acids; after deamidation of Gln and Asn by the action of Nt-amidase enzymes NTAN1 and NTAQ1 or Cys oxidation, these residues are converted to secondary destabilizing amino acids and consequently substrates for ATE1 (Graciet and Wellmer, 2010). It is important to note that in all cases, these amino acids can act as destabilizers only if they are exposed at the N-terminus. To serve as a degradation substrate, the protein must loose the first Met by the action of a Met-aminopeptidase (MAP) (Bradshaw *et al*., 1998). This reaction can occur during translation if the second amino acid is non-bulky, or by selective cleavage by cellular endoproteases.

It is interesting to note that N-recognins have diversified in different ways in plants and animals. Yeast cells encode a single protein, UBR1, which recognizes basic and hydrophobic N-terminal residues (Sriram *et al*., 2011). In contrast, two types of structurally unrelated PRTs are known in plants, PRT1 and PRT6, which recognize hydrophobic and basic residues, respectively. Animals encode different UBR isoforms, but similar to yeast N-recognins, these proteins can bind to both types of primary destabilizing residues (Sriram *et al*., 2011).

## Plant ATEs and their evolutionary relationship with other ATEs

Studies comparing the N-end rule pathway of bacteria, fungi, animals and plants have shown that this is an ancient protein degradation pathway that likely evolved before the advent of the UPS in eukaryotes (Graciet *et al*., 2006; Xia *et al*., 2008). Supporting this conclusion is the evidence of a common hierarchical organization and conservation of the enzymatic components involved in N-terminal protein modification, such as ATE and Nt-amidase enzymes, during evolution (Graciet and Wellmer, 2010). Dissection of plant N-end rule pathway components as well as identification of two *Arabidopsis* Nt-amidases mediating recognition of tertiary destabilizing Nt-amino acids Asn and Gln have shown that the N-end rule pathway in plants is very similar to that in animals, highlighting a possible evolutionary common origin (Graciet and Wellmer, 2010; Graciet *et al*., 2010). The steps related to protein degradation, however, likely evolved after plant and animal divergence, as suggested by the differences in PRTs and UBR N-recognins.

**Figure 1:**
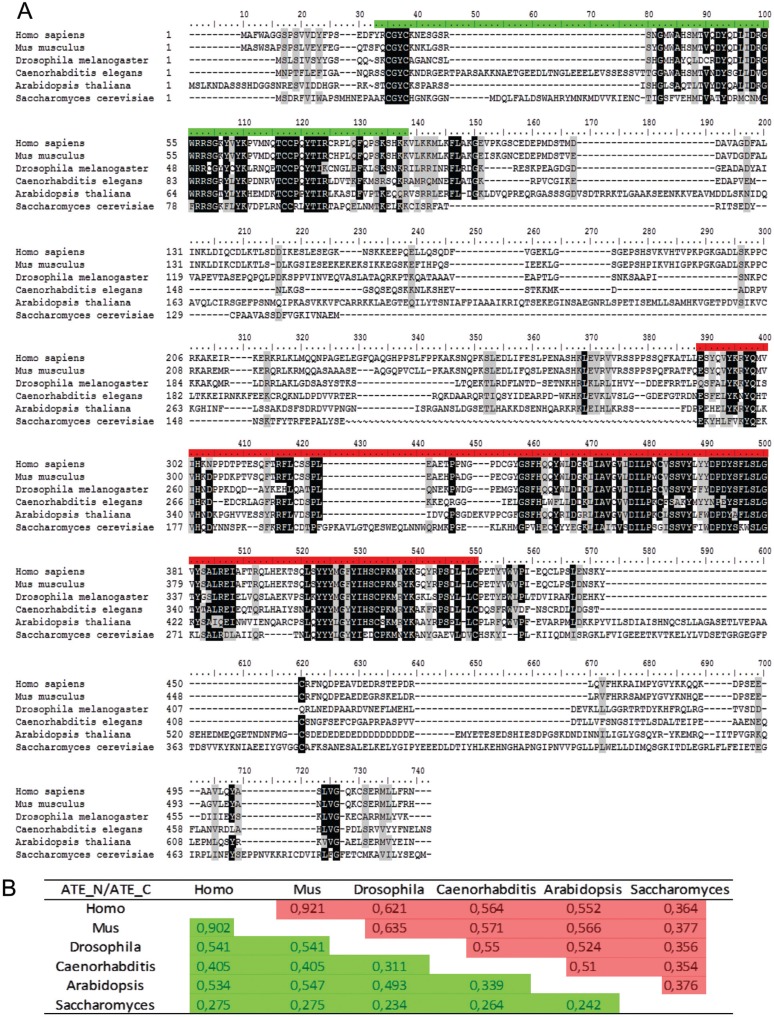
ATE protein sequences across distinct kingdoms. A) Alignment of ATE full-length protein sequences from *Homo sapiens* (NP_001001976.1), *Mus musculus* (NP_038827.2), *Drosophila melanogaster* (NP_477394.3), *Caenorhabditis elegans* (NP_492549.2), *Arabidopsis thaliana* (At5g05700) and *Saccharomyces cerevisiae* (AJS07672.1) are shown. Green and red bars represent ATE-N and ATE-C terminal domains, respectively, as indicated by PFAM analysis. Columns with 100% and > 80% amino acid identity are shaded in black and gray, respectively. B) Identity values of the ATE-N (green) and ATE-C (red) domains between different species.

ATE protein sequences contain two Pfam domains named ATE-N (PF04376) and ATE-C (PF04377), which are located at N- and C-termini, respectively (Figure 1). Comparative alignment and identity analysis of ATE proteins from human, mouse, fruit fly, yeast and *Arabidopsis* have revealed high conservation in domain regions but variability in inter-domain sequence and size (Figure 1A, B). Interestingly, the observed identity of more than 60% between the C-terminal ATE domains of mammals and fruit flies may reflect conserved functions or interaction partners in these taxa (Figure 1B). It is also remarkable that the plant ATE C-domain shares 50% identity with the same domain from *C. elegans* to human, demonstrating strong protein conservation among multicellular eukaryotes.

**Figure 2:**
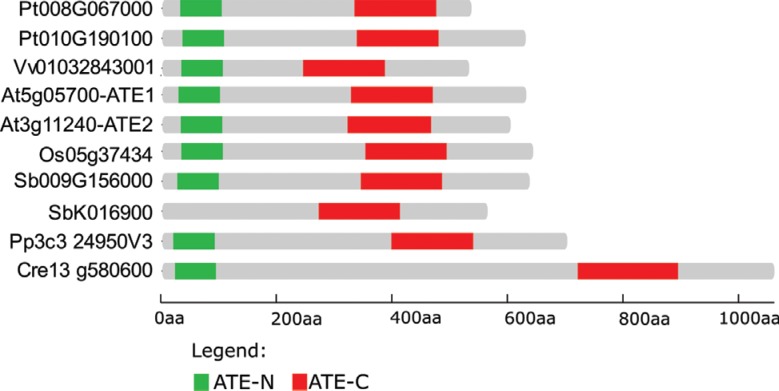
Domain location of *ATE* protein sequences among plant species. ATE coding sequences from distinct plant species, such as the eudicots *Populus trichocarpa*, *Arabidopsis thaliana* and *Vitis vinifera*, monocots *Sorghum bicolor* and *Oryza sativa*, moss *Physcomitrella patens* and green alga *Chlamydomonas reinhardtii*, were extracted from Phytozome V11.0. The full-length protein schemes were generated by Gene Structure Display Server 2.0 software; shown are the ATE domains identified by Pfam v29.0.

Plant evolutionary analysis has identified *ATE* orthologous genes from the green alga *Chlamydomonas reinhardtii* to angiosperms (Figure 2). In general, only one *ATE* gene is detected in a given plant species, with the two conserved ATE domains located at the N- and C-termini (Figures 1, 2). Some species, such as *Arabidopsis*, *Populus* and *Sorghum*, have experienced gene duplication. However, *Sorghum* appears to have generated a paralog gene that contains only the ATE-C domain and is likely not a functional arginyltransferase.

Recently, it was demonstrated that ATE protein abundance is spatially and temporally regulated by hormones and light and is highly abundant in meristematic cells from the moss *Physcomitrella patens* (Schuessele *et al*., 2016). This work also showed that arginylation is necessary for moss gametophyte development, which is not observed in flowering plants. These findings support the conservation of N-end rule pathway components in land plants, even though the regulated processes may have diverged during evolution.

## Plant ATE biological functions

Arabidopsis encodes two closely related *ATE* genes, *AtATE1* (At5g05700) and *AtATE2* (At11240), which have at least partially redundant functions. *ATE1* was first functionally investigated by Yoshida *et al*. (2002) in the mutant *delayed leaf senescence1* (*dls1*), which exhibits extremely slow age-dependent, dark-induced leaf senescence. Such a phenotype was linked to a disrupted *ATE1* in *dls1*. Years later, Holman *et al*. (2009) showed that the N-recognin E3 ligase PRT6 and *AtATE1* and *AtATE2* are involved in seed germination controlled by abscisic acid. A double mutant for *AtATE1* and *AtATE2* (*ate1.ate2*) displays lost sensitivity to this hormone and consequently uncontrolled seed germination and establishment. At the end of the same year, Graciet *et al*. (2009) showed that apical dominance, stem elongation and regulation of leaf morphology were also linked to the N-end rule pathway, as demonstrated by analyzing *AtATE1* and/or *AtATE2* T-DNA insertion mutants (Graciet *et al*., 2009; Graciet and Wellmer, 2010). The arginylation branch of the N-end rule pathway is also responsible for repressing expression of the meristem-promoting brevipedicellus (*BP*) gene during leaf development, acting in a redundant way with the asymmetric leaves 1 (AS1) transcription factor complex, a known negative regulator of *BP* expression (Graciet *et al*., 2009). The R-transferase target in this mechanism, however, has not yet been identified. Another important discovery by Graciet *et al*. (2009) was that the N-recognin PRT6 is the E3 ligase N-recognin of plants and acts downstream of ATE.

One of the most studied and understood signaling pathways in plants controlled by arginylation is that involving the ethylene responsive transcription factor VII (ERFVII). It was observed that *Arabidopsis ate1/ate2* or *prt6* mutants could not degrade ERFVII, and as a consequence showed increased expression of hypoxia–responsive genes involved in fermentation and sugar consumption even under oxygen-rich conditions (Gibbs *et al*., 2011; Licausi *et al*., 2011). The mechanism responsible for ERFVII regulation was suggested by previous studies on mammalian ATE1, which revealed that these enzymes can recognize oxidized N-terminal Cys residues as substrates for arginylation. The occurrence of this was later demonstrated in plants, with oxidized Cys also acting as a tertiary destabilizing N-terminal residue (Graciet *et al*., 2010). Gibbs *et al*. (2011) demonstrated *in vitro* that all members of *Arabidopsis* group VII ERFs are N-end rule substrates that function as sensors of molecular oxygen via oxidation of the tertiary destabilizing cysteine residue. *Arabidopsis* has five ERFVII proteins: hypoxia-responsive ERF 1 and 2 (HRE1 and HRE2) and three more proteins related to apetala 2.2: RAP2.2, RAP.2.3, RAP2.12 (Nakano *et al*., 2006). Alignment of the five *Arabidopsis* VII ERFs revealed that all share the same five first N-terminal residues (Gibbs *et al*., 2011, 2015), with the Nt-MCGGAII/L domain being highly conserved in flowering plants (Licausi *et al*., 2011). Moreover, all five ERFVII proteins from *Arabidopsis* accumulate under conditions of low oxygen and are destabilized by the presence of oxygen. Oxidation of the N-terminal Cys residue is catalyzed by plant cysteine oxidases (PCOs) under specific conditions in which the Nt-Met residue is removed by MAP activity. Therefore, arginylation is directly involved in the mechanism that enables this transcription factor family to function as a sensor of hypoxia in plants (Gibbs *et al*., 2011; Licausi *et al*., 2011).

Another important biological process mediated by Cys arginylation was demonstrated years later. Gibbs *et al*. (2014) showed that group VII ERFs are also destabilized by nitric oxide (NO), and the same type of regulation was previously demonstrated in animal cardiovascular development. The target protein is the MC-initiating (Nt-MetCys) regulator of G-protein signaling (RGS) protein. RGS is destabilized in the presence of NO and oxygen, which mediate conversion of Nt-Cys to Cys sulfonic acid, making RGS a substrate for ATE and the N-end rule pathway (Hu *et al*., 2005; Jaba *et al*., 2013). Gibbs *et al*. (2014) found that the same mechanism regulates ERFVII stability in plants. In the presence of oxygen and/or NO, ERFVII degradation leads to hypocotyl growth inhibition, seed germination, photomorphogenesis, stomatal closure, and hypoxia-responsive transcriptional repression. Under hypoxia, however, accumulation of group VII ERFVs promotes hypocotyl growth, seed dormancy, photomorphogenesis repression, stomatal opening, and anaerobic-responsive gene expression (Gibbs *et al*., 2015). Together, these studies suggest that group VII ERFs function as a central hub for the perception of oxygen and NO and that the N-end rule pathway is an important integrator of gas signaling in plants (Gibbs *et al*., 2014). The regulatory role can be even broader, as ERFVII arginylation can also respond to other signals such as ethylene (Gibbs *et al*., 2015).

By studying water submergence and starvation stress responses in N-end rule pathway mutants, Riber *et al*. (2015) observed that these mutant plants were generally more tolerant to starvation conditions, such as prolonged darkness or water submergence. Interestingly, increased tolerance was a direct consequence of the impaired N-end rule pathway rather than up-regulation of the expression of genes involved with sugar consumption and fermentation (Riber *et al*., 2015). This work showed the potential of regulating the ATE branch of the N-end rule pathway to generate stress-resistant plant variants.

The identification of ATE arginylation targets is an intriguing area of research and can reveal new biological processes regulated by the N-end rule pathway. Based on sequence analysis only, a large number of proteins appear to be potential substrates for ATE-mediated destabilization. However, the experimental identification and characterization of ATE substrate proteins remain technically challenging. The best characterized targets proteins are mammalian RGS proteins, especially RGS4, RGS5 and RGS16, and plant group VII ERFs (Hu *et al*., 2005; Gibbs *et al*., 2011; Licausi *et al*., 2011; Bailey-Serres *et al*., 2012; Lee, *et al*., 2012; Gibbs *et al*., 2014). As mentioned above, both RGS and ERFVII substrates have a Cys as the second Nt residue which, after oxidation, acts as a tertiary destabilizing residue dependent on ATE arginylation (Gibbs *et al*., 2011; Licausi *et al*., 2011; Bailey-Serres *et al*., 2012; Lee *et al*., 2012). During oxygen-limiting conditions, ERFVII and RGS accumulate, leading to transcription of hypoxia-response genes in plants (Gibbs, *et al*., 2011; Licausi *et al*., 2011; Gibbs *et al*., 2014) and decreased cardiomyocyte proliferation by RGS accumulation and downregulation of G protein signaling in mammals (Lee *et al*., 2012).

The proteins' resistance to *Pseudomonas syringae* 1-interacting protein 4 (RIN4), ethylene response factor 72 (EBP) and vernalization 2 (VRN2) have been identified as putative ATE substrates by transcriptome analysis of *ate1.ate2* mutants. However, their modification by ATE has not been shown at the molecular level (Gibbs *et al*., 2011).

Mass spectrometry approaches are being developed to screen for arginylated proteins and to reveal new cellular processes controlled by ATE modification. To identify plant ATE substrates, Majovsky *et al*. (2014) analyzed plant proteomes from *A. thaliana ate1*, *ate2*, *prt1* and *prt6* knockout mutants, and new putative substrates of ATE were identified among Nt Met-Cys proteins. The relative abundance of methylesterase 10 (MES10), nucleoside diphosphate kinase family protein (NDPK1), and two asparagine synthetases (ASNs) was augmented in *ate1/ate2* mutants. The MES10 protein hydrolyzes methyl salicylate to salicylic acid, NDPK1 plays a role in the response to reactive oxygen species (ROS) stress, and ASNs are components of the L-asparagine biosynthesis pathway (Peeters *et al*., 2000; Fukamatsu *et al*., 2003; Yang *et al*., 2008). Other putative substrates found by Majovsky *et al*. (2014) that do not have a Nt-Met-Cys were glyceraldehyde 3-phosphate dehydrogenase subunits GAPA, GAPA2 and GAPB, the granulin repeat cysteine protease family protein (RD21), glucoside (GGD), thioglucoside glucohydrolases (TGG1 and TGG2), two glycine-rich proteins (GRPs), cold circadian rhythm (CCR1 and CCR2), RNA-binding 1 and 2 and nicotinamide adenine dinucleotide-dependent malic enzyme (NAD-MED2). The functional implication of these modifications awaits experimental validation.

**Figure 3:**
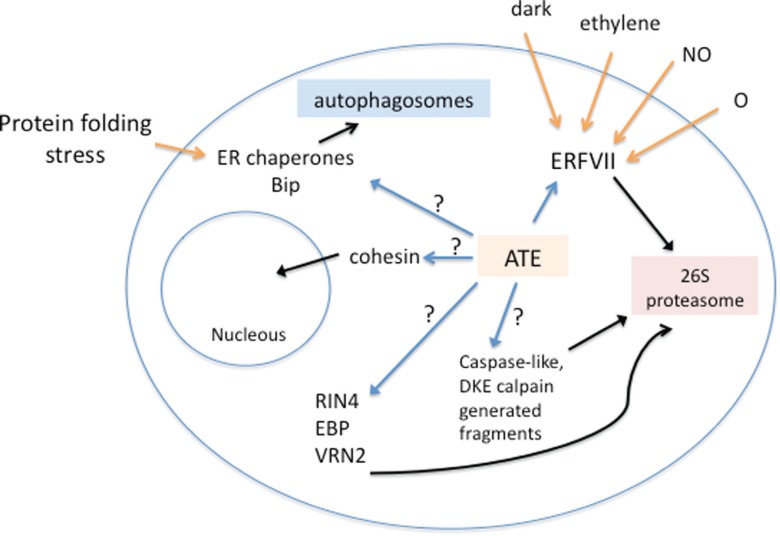
A proposed model of biological processes regulated by ATE arginylation in plants. Plant ATE putative and confirmed substrates are indicated by blue arrows. Black arrows show the possible fates of N-terminal arginylated proteins. Yellow arrows indicate external signals known to induce N-terminal modifications necessary for ATE substrate recognition, such as ERFVII Cys oxidation induced by oxygen and NO or cytosol translocation of N-terminally cleaved ER chaperones induced by protein folding stress. In this model, we include the putative targets and signaling pathways observed in mammalian cells that have not been tested in plant cells, as indicated by question marks.

Other interesting roles for mammalian ATEs are emerging and could point to new functions in plants. Recently, Cha-Molstad *et al*. (2015a) showed that in human cells, ATE1 mediates N-terminal arginylation of binding immunoglobulin protein (Bip) and possibly other endoplasmic reticulum (ER)-residing chaperones. Under certain stress conditions, such as the presence of dsDNA in the cytoplasm or proteasome inhibition, Bip was directed to the cytosol, where it was arginylated by ATE. Further experiments showed that Nt-Arg residues function as a determinant of autophagic delivery to autophagosomes by acting as an activating ligand of p62, an important component of the autophagy pathway that interacts with degradation targets and drives autophagosome formation by recruiting other components of the pathway (Yamano and Youle, 2013; Cha-Molstad *et al*., 2015a,b). Intriguingly, ATE was also reported to act in mammals directing the degradation of caspase- and calpain-generated fragments of cohesin (Rai and Kashina, 2005; Piatkov *et al*., 2012, 2014). Nonetheless, the involvement of plant ATEs in the autophagy pathway and in the degradation of caspase-like and/or calpain-generated fragments in plants remains elusive. Combining these putative ATE mechanisms with those already studied in plants, we propose a model of biological processes regulated by ATE arginylation in plants (Figure 3).

## Concluding remarks

Although important efforts have been made towards an understanding of the role of ATE/N-end rule functions in plants, many open questions remain, for example, the functions of different isoforms of ATE in plants, how ATE activity is regulated, and the identification of interaction partners. It is possible that plant ATEs can also arginylate internal amino acids of proteins and that this type of modification has other signaling functions.

The discovery of the physiological substrates of R-arginylation is essential for understanding N-end rule functions. In addition to its biological importance, elucidating some of the processes regulated by the N-end rule pathway in plants is relevant for biotechnological purposes, as this pathway has a direct impact on developmental processes and tolerance to starvation and/or abiotic stress.
